# Giant cell temporal arteritis: a clinicopathological study with emphasis on unnecessary biopsy

**DOI:** 10.3389/fopht.2023.1327420

**Published:** 2023-12-06

**Authors:** Hind M. Alkatan, Fawziah AlMana, Azza M. Y. Maktabi

**Affiliations:** ^1^ Department of Ophthalmology, College of Medicine, King Saud University, Riyadh, Saudi Arabia; ^2^ Department of Pathology, College of Medicine, King Saud University, Riyadh, Saudi Arabia; ^3^ King Saud University Medical City, King Saud University, Riyadh, Saudi Arabia; ^4^ College of Medicine, Alfaisal University, Riyadh, Saudi Arabia; ^5^ Pathology and Laboratory Medicine Department, King Khaled Eye Specialist Hospital, Riyadh, Saudi Arabia

**Keywords:** giant cell arteritis, temporal artery, ischemic optic neuropathy, temporal artery biopsy, atherosclerosis, optic nerve

## Abstract

**Introduction:**

Temporal artery (TA) biopsy is commonly used for the diagnosis of giant cell arteritis (GCA). However, a positive biopsy is no longer mandatory for diagnosis. This study aims to correlate the histopathological findings of TA biopsies in suspected cases of GCA to the clinical presentation in an ophthalmic tertiary eye care center to draw useful conclusions and advocate the possible implementation of guidelines for TA biopsy.

**Methods:**

Data was collected from patients’ medical records including, demographics, clinical data, and histopathological findings and diagnosis. The 2022 American College of Rheumatology/ European Alliance of Associations for Rheumatology (ACR/EULAR) criteria have been used and partially adopted as a guide to compare the variables between TA biopsy-positive and negative groups as well as the TA biopsy-positive group and the group of patients with TA biopsy showing atherosclerosis.

**Results:**

Out of the total 35 patients who underwent a TA biopsy during the period of 23 years, 22.9% of patients had histopathological findings consistent with GCA and 42.9% had TA atherosclerotic changes, while the remaining 34.3% had histologically unremarkable TA. The mean age of all patients was 66 ± 10.9 years. Slightly more than half were females (54.3%) and the remaining were males (45.7%). In the group with positive TA biopsies, the mean age was 71 ± 8.4 years with a higher female predominance (female-to-male ratio of 5:3). The mean diagnostic clinical score used in our study was higher (7.5 ± 2.33) in the GCA-positive group when compared to the other groups with statistical significance (mean of 4.85 ± 2.01 in patients with overall GCA-negative biopsies and 5.13 ± 2.10 in the group with atherosclerosis). Other three clinical variables that were found to be statistically significant in the GCA biopsy-positive group were scalp tenderness, jaw claudication, and optic nerve pallor.

**Discussion:**

The mean age (71 ± 8.4 years) and the female predominance of GCA in our group of patients with positive TA biopsy (62.5%) was like other reports. In our study 22.9% of performed TA biopsies over the period of the study were positive confirming the diagnosis of GCA on histological exam, which was similar to another report and is considered to be relatively low. The incorporation of increased clinically focused assessments and algorithms, with the aid of the ACR/EULAR criteria, may decrease the frequency of TA biopsies that carries unnecessary cost and risk of procedure-related morbidity. We highly recommend applying the age of ≥ 50 years as an initial criterion for diagnosis, followed by the consideration of the statistically significant clinical features: scalp tenderness, jaw claudication, and optic nerve pallor.

## 1 Introduction

Giant Cell Arteritis (GCA) is a large-cell vasculitis that preferentially affects large to medium-sized arteries. GCA most commonly develops in those over the age of 50 with a peak incidence in the eighth decade of life and a slight female predominance (Female to male ratio of 3:1). Globally, GCA incidence varies. The highest recorded incidence is found in Northern Europe (44/100,000 persons over 50) and the lowest in Sothern Asia (0.3/100,000). In the Middle East, the incidence ranges from 4.9 to 11.3 per 100,000 persons over 50 (1). It has a particular affinity for the branches of the external and internal carotid arteries, including the temporal artery, hence the term temporal arteritis (2). Genome-wide association studies have found that polymorphisms in the HLA-DR region are highly linked to the GCA autoimmune response (3). This response includes interactions between the innate and adaptive immune system, that trigger granulomatous inflammation in the tunica media and destruction of the arterial wall’s elastic tissue. As a result, temporal artery (TA) biopsies have long been the gold standard for diagnosing GCA. However, according to the American College of Rheumatology (ACR), a positive TA biopsy is not mandatory for the diagnosis of GCA (4). The 2022 American College of Rheumatology/European Alliance of Associations for Rheumatology (ACR/EULAR) have also developed classification criteria as a guide for diagnosing GCA (5). Therefore, in a setting of a tertiary eye center with limited or no access to a highly specialized rheumatology unit (to aid in the clinical diagnosis), unnecessary TA biopsies may be performed. The aim of this study is to correlate the histopathological findings of TA biopsies in our eye center to the clinical presentation. This will emphasize the importance of developing proper clinical and diagnostic criteria and highlight possible guidelines for TA biopsy performance in suspected cases.

## 2 Materials & methods

This is a retrospective study of all TA biopsies that were received by the Pathology and Laboratory Medicine Department, King Khaled Eye Specialist Hospital (KKESH) during the period: June 2000 to June 2023. The corresponding patients’ medical records were reviewed to collect demographics, and clinical information including the clinical symptoms, signs, duration of presentation, and relevant laboratory investigations as well as biopsy information (date and results), using a special data collection sheet. The initial histopathological data included a total of 38 unilateral TA biopsies received over the above study period. Demographic and clinical data were not available in three patients thus, they were excluded. 35 cases have been included for analysis in this study.

The 2022 American College of Rheumatology/European Alliance of Associations for Rheumatology (ACR/EULAR) classification criteria have been used as a guide to compare the TA biopsy-positive group and TA biopsy-negative group (**Supplementary Material**). We have adopted a GCA diagnostic score assigned to each patient that excludes laboratory investigations and the scoring for the TA biopsy itself. The diagnostic scoring system includes the following: morning stiffness in the shoulders/neck, sudden visual loss, jaw or tongue claudication, new temporal headache, scalp tenderness, and abnormal examination of the temporal artery. For each positive sign or symptom, two points were given, except for sudden visual loss, for which three points were given. The available erythrocyte sedimentation rate (ESR) was used as a separate variable, independent of the modified scoring mentioned above. C-reactive protein (CRP) measurement was not done for most patients and thus, was not included as a variable in our analysis.

The variables used in our baseline analysis to calculate the aforementioned diagnostic score are highlighted in **Table 1**. According to the ACR/EULAR guidelines, which include laboratory investigations, imaging (ultrasound and PET) results, and biopsy results, any score greater than or equal to six, is needed for the classification of giant cell arteritis (5). Since we did not have availability for all tests included in the guidelines, and considering our goal of comparing the diagnostic clinical criteria between the two patient groups to justify performing a TA biopsy, we did not enforce the cut-off scoring of six on our cases. Rather, we have utilized the clinical items in the ACR/EULAR guidelines as variables, to indicate by analysis, the potentially important items that showcase a statistically significant difference between the two groups. As a result, these variables can aid in the pre-biopsy clinical judgment and diagnosis of GCA, and can guide ophthalmologists in their decision to proceed further with a TA biopsy or not. Other significant signs and symptoms that were recorded apart from the ones used in the ACR/EULAR guidelines, were noted as well in the same table.

**Table 1:** Comparison between the GCA biopsy-positive and biopsy-negative groups.

Variables		Biopsy Results				*p* (<0.05)
		GCA Positive (n = 8)		GCA Negative (n= 27)		
		Frequency	%	Frequency	%	
Components of Diagnostic Score	Sudden visual loss	8	100	23	85.2	0.553
	New temporal headache	5	62.5	19	70.4	0.685
	Jaw or tongue claudication	3	37.5	2	7.4	0.067*
	Morning stiffness in shoulders and/or neck	1	12.5	3	11.1	>0.999
	Scalp tenderness	7	87.5	5	18.5	**0.001**
	Abnormal temporal exam	2	25.0	4	14.8	0.602
Corresponding Diagnostic Score (mean ± SD)**		7.5 ± 2.33		4.85 ± 2.01		**0.003**
Neck pain		2	25.0	3	11.1	0.568
Sweats		0	0.0	4	14.8	0.553
Generalized weakness		2	25.0	3	11.1	0.568
Optic nerve changes		6	75.0	21	77.8	>0.999
Optic nerve pallor		6	75.0	8	29.6	**0.039**
Elevated ESR (≥50 mm/hr)		7	87.5	22	81.5	>0.999

GCA, Giant cell arteritis * This p-value was re-assessed by comparing the eight GCA-positive patients to the 12 patients with normal TA biopsy findings & was found to be statistically significant with p = 0.049. **Defined as all of the items mentioned above the score (i.e., sudden visual loss, new temporal headache, jaw or tongue claudication, morning stiffness in shoulders and/or neck, scalp tenderness, abnormal temporal artery exam).

Bold values are statistically significant P values.

Data was analyzed using “SPSS” Version 20.0. Categorical variables are presented as percentages and frequencies and are compared using the Fisher’s Exact Test. Clinical scores are presented as a mean ± standard deviation and were compared using the Independent Samples T-test. The *p* values mentioned are all two-sided, and *p* values <0.05 were considered to denote statistical significance. This study was prepared in accordance with the ethical standards of the human ethics committee (HEC) at KKESH and expedited approval as a retrospective study from the HEC/IRB of the Research department in accordance with the Helsinki Declaration.

## 3 Results

Thirty-five patients who underwent TA biopsy were included for analysis based on their respective biopsy results and histopathological diagnoses. Out of these, 8/35 (22.9%) had histopathological findings consistent with GCA (with typical features of active arteritis in 7/8 and one (1/8) burnt-out case). The remaining 27/35 (77.1%) biopsies did not confirm the diagnosis of GCA and were either histologically unremarkable in 12/35 (34.3%) or consistent with atherosclerosis in 15/35 (42.9%). The results of this study have been reported for the entire patient group, for the positive biopsies, and the negative biopsies, respectively. Two sets of analysis results were performed, one to compare the two originally defined groups of eight biopsy-positive patients to the 27 biopsy-negative, and the second to compare the eight biopsy-positive cases to the 15 atherosclerotic cases, excluding patients with normal TA biopsy.

### 3.1 Demographic Results

The mean age of all patients was 66 ± 10.9 years. Slightly more than half (19/35) were females (54.3%). The remaining 16/35 were males (45.7%). In regards to nationality, 97.1% (34/35) of patients were Saudis and 2.9% (1/35) were non-Saudis. Out of all Saudi patients (34), 52.9% (18/34) originated from the Central province of the country.

In the group with positive TA biopsies, the mean age of patients was 71 ± 8.4 years with a higher female predominance in 62.5% of the cases and a female-to-male ratio of 5:3. The patients in this group were all Saudi nationals. In the group with negative TA biopsies, the mean age was 65 ± 11.2 years with almost equal gender distribution: 14/27 females (51.9%) and 13/27 males (48.1%). Non-Saudis constituted 3.7% (1/27) of the patients.

### 3.2 Histopathology

**Figure 1:**
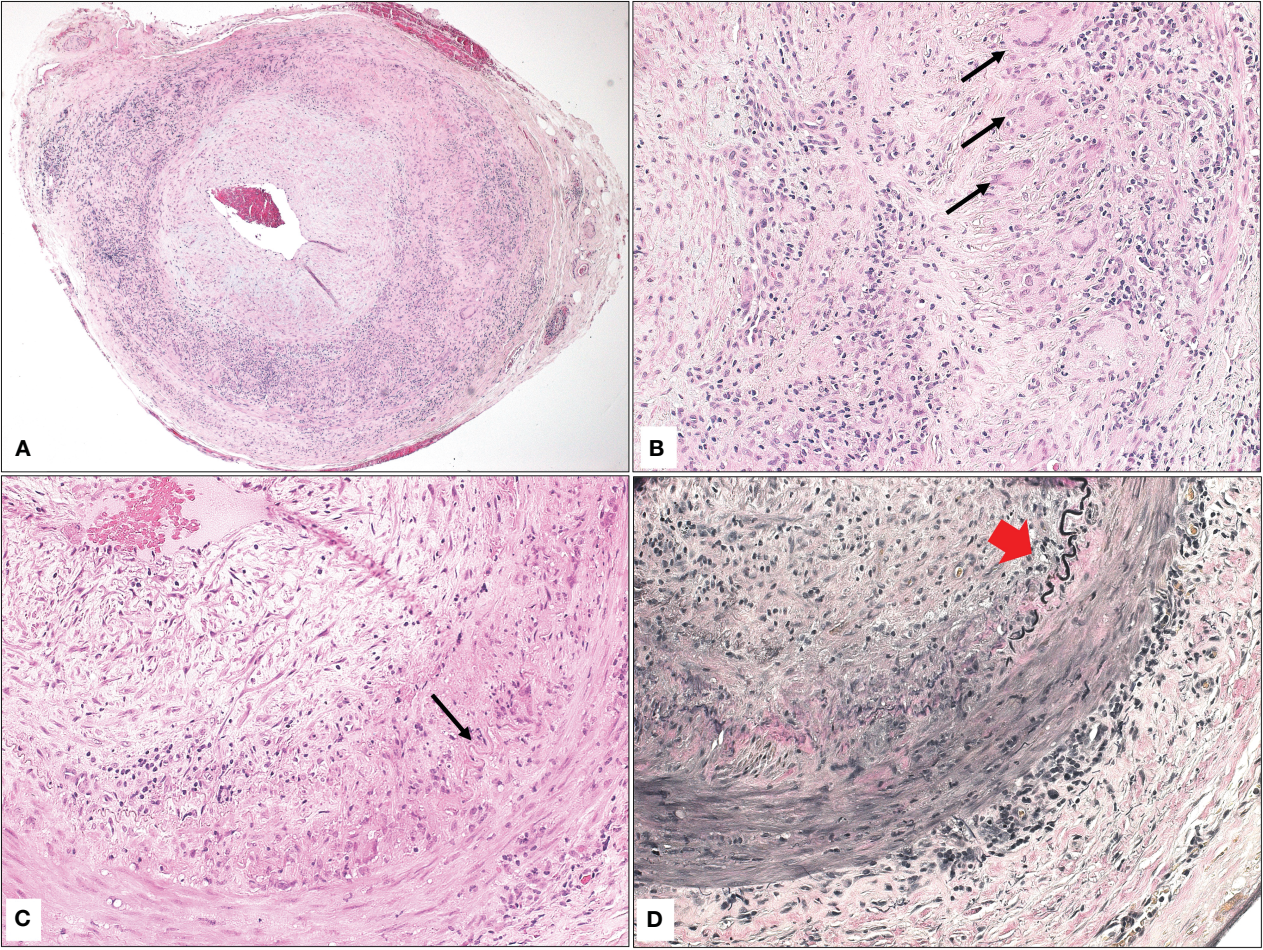
**(A)** The histopathological appearance of a temporal artery with narrowed lumen, atrophic media, interrupted elastic lamina, and granulomatous inflammation consistent with the diagnosis of giant cell arteritis (Original magnification x 50 Hematoxylin & eosin). **(B)** Higher power of the typical area of granulomatous inflammation with multiple giant cells (black arrows) in the area of interrupted elastic lamina (Original magnification x 400 Hematoxylin & eosin). **(C)** Similar findings in another temporal artery biopsy with interrupted elastic lamina (black arrow), granulomatous inflammation, and giant cell formation (Original magnification x 200 Hematoxylin & eosin). **(D)** The area of interrupted elastic lamina (red arrowhead) is clearer now after special staining (Original magnification x 200 Elastin).

The TA biopsies were all grossly examined with measurements of the temporal artery dimensions (length and diameter). All biopsies measured 2.0 cm or more in length and were serially sectioned and submitted “en toto.” In 7/8 of the positive cases, the TA showed typical interrupted internal elastic lamina with granulomatous reaction in the same areas of interruption defined as a collection of histiocytes with formation of variable numbers of giant cells (**Figures 1A, B**). Secondary findings included narrowing of or occluded lumen with thickened intima and minor degenerative changes in the media. In addition, a similar granulomatous reaction was observed within the adventitia in the remaining burnt-out case (1/8). The interruption of the internal elastic lamina was nicely demonstrated using elastin stain (**Figures 1C, D**). In contrast, 15/27 of the negative biopsies (55.6%) displayed atherosclerotic changes with narrowing of the lumen, thickened intima, atrophy of the media, and calcific plaques. The remaining 12/27 (44.4%) demonstrated histologically unremarkable TA biopsies.

### 3.3 Clinical results

The clinical/diagnostic score we have adopted from the ACR/EULAR criteria was utilized to assign each patient a clinical score. In the eight patients with positive GCA on histopathology, scores ranged from five to 12, with a mean value of 7.5 ± 2.33, as opposed to a mean of 4.85 ± 2.01 in patients with GCA-negative biopsies (*p*-value = 0.003). The variables used to calculate this score as well as additional clinical signs and symptoms are summarized in **Table 1**, with a calculated level of statistical significance for each. The clinical features that were found to be significantly more common in the GCA biopsy-positive group were scalp tenderness and optic nerve (ON) pallor (*p =* 0.001 and *p* = 0.039, respectively). Moreover, the *p*-value for jaw and/or tongue claudication was found to be close to statical significance (*p =* 0.067) and thus, was re-assessed by comparing the eight GCA-positive patients to the 12 patients with normal TA biopsy findings (excluding the ones with atherosclerosis). As a result, this variable was found to be statistically significant with *p* = 0.049.

Regarding the lab results, elevated ESR (≥50 mm/hr) was seen in 7/8 (87.5%) and 22/27 (81.5%) of GCA-positive and GCA-negative cases, respectively.

Similar observations and statistically significant differences were found when comparing the eight GCA-positive patients and the 15 patients with atherosclerotic changes on TA biopsy (summarized in **Table 2**) in terms of the adopted clinical score, the presentation with scalp tenderness, and the presence of ON pallor on fundoscopic examination (*p* values of 0.022, 0.006, and 0.023 respectively).

**Table 2:** Comparison between the biopsy-positive and atherosclerotic changes groups.

Variables		Biopsy Results				*p* (<0.05)
		GCA Positive (n = 8)		Atherosclerotic Changes (n= 15)		
		Frequency	%	Frequency	%	
Components of Diagnostic Score	Sudden visual loss	8	100	14	93.3	>0.999
	New temporal headache	5	62.5	10	66.7	>0.999
	Jaw or tongue claudication	3	37.5	2	13.3	0.297
	Morning stiffness in shoulders and/or neck	1	12.5	1	6.7	>0.999
	Scalp tenderness	7	87.5	3	20.0	**0.006**
	Abnormal temporal exam	2	25.0	2	13.3	0.589
Corresponding Diagnostic Score (mean ± SD)*		7.5 ± 2.33		5.13 ± 2.10		**0.022**
Neck pain		2	25.0	1	6.7	0.269
Sweats		0	0.0	3	20.0	0.526
Generalized weakness		2	25.0	2	13.3	0.589
Optic nerve changes		6	75.0	10	66.7	>0.999
Optic nerve pallor		6	75.0	3	20.0	**0.023**
Elevated ESR (≥50 mm/hr)		7	87.5	14	93.3	>0.999

GCA, Giant cell arteritis. * Defined as all of the items mentioned above the score (i.e., sudden visual loss, new temporal headache, jaw or tongue claudication, morning stiffness in shoulders and/or neck, scalp tenderness, abnormal temporal artery exam).

Bold values are statistically significant P values.

## 4 Discussion

Temporal artery (TA) biopsy remains a chief diagnostic tool for GCA. However, its relatively invasive nature and variable sensitivity make its routine implementation difficult (6). Therefore, unnecessary TA biopsies can be avoided with the usage of clinically oriented diagnostic tools. In Saudi Arabia, GCA has been found to be consistently of low incidence, not unlike other Middle Eastern and Mediterranean countries (7). A prior retrospective analysis conducted at KKESH, found that 6.8% (7/102) of patients who underwent TA biopsy between 1983 – 2004, had biopsy-positive GCA (8). In our study, a higher proportion of biopsies (22.9%) performed over 23 years (2000 – 2023) conveyed positive GCA on histological exam. Kaltsonoudis et al. similarly described that only 20% of TA biopsies performed at their institution over 17 years (2000 – 2017) were positive for GCA (9). We still believe that the number of negative biopsies at our institution is relatively high and consequently, some of these biopsies may be considered unjustifiable. This emphasizes the need for better clinical assessment before the TA biopsy is performed.

Almost all patients with GCA biopsy-positive (7/8) had typical active arteritis findings on histopathological examination, affecting all layers of the arterial wall with one burnt-out case with residual typical granulomatous arteritis involving the adventitia. Hernández-Rodríguez et al. reported that biopsies with more extensive infiltration into the arterial wall were increasingly associated with jaw claudication and scalp tenderness, in contrast to those with exclusive adventitial involvement (10). Owing to the small number of biopsy-positive cases in this study, such correlation is not expected to be reliable. However, while there were appreciable differences between the frequency of presenting clinical signs, scalp tenderness was the most important sign with a statistically significant difference between biopsy-positive and biopsy-negative groups (*p* = 0.001), and between the biopsy-positive and atherosclerosis group (*p* = 0.006). Molina-Collada et al. and van Nieuwland et al. both reported similar findings in their respective investigations, highlighting the importance of this sign in clinical assessment (11, 12). To add, new-onset jaw claudication is one of the most common symptoms reported by GCA patients (13). In our study, jaw claudication was seen in 3/8 (37.5%) of biopsy-positive patients versus 2/27 (7.4%) of biopsy-negative patients. Even though the *p*-value (*p* = 0.067) was not statistically significant between the two groups, one must keep in mind that the GCA-negative group included normal TA biopsies as well as TA biopsies showing atherosclerosis. Therefore, upon recalculation of the p value by comparing the eight GCA-positive cases to the 12 cases with normal TA biopsy (excluding atherosclerotic TA biopsies), statistical significance was found with *p =* 0.049 as per the footnote in **Table 1**. Thus, there is increasing evidence to believe that jaw claudication is an important sign with the more evenly assigned grouping. Lecler et al. demonstrated a more evenly assigned patient grouping, with 25 GCA-positive and 20 GCA-negative patients. In their study, jaw claudication showed an even higher statistical significance of *p* = 0.001, occurring in 64% of GCA-positive patients while none of the GCA-negative patients showed such a sign (14). Similarly, none of our patients with normal TA biopsies experienced jaw claudication. Our conclusion is further supported since the presence of this sign was not found to be statistically significant when comparing the eight GCA-positive patients to the 15 patients in the atherosclerosis group alone, as seen in **Table 2**.

Regarding the presenting symptoms, it has been reported that visual disturbances, most commonly sudden visual loss, are a hallmark of giant cell arteritis (8, 9, 15). In our study, all biopsy-positive patients experienced some degree of subjective sudden visual loss as opposed to the 85.2% (23/27) of biopsy-negative patients. However, since our study was conducted in an eye tertiary care center, where patients are referred mostly for visual disturbances, the biopsy-negative group will include patients who have experienced visual loss due to other causes. These patients have been likely referred to the center mainly because of their visual symptoms rather than a constellation of systemic symptoms consistent with GCA (8).

GCA’s inflammatory nature may trigger the occlusion of orbital arteries, causing optic nerve pallor (16). The arteritic ischemic optic neuropathy that develops in GCA has been proven to manifest acutely as optic nerve pallor, illustrating fundoscopy’s vital nature in the diagnosis of patients with potential GCA (17). On fundoscopy, ON pallor was one of the presenting signs showing statistical significance in the two previously mentioned comparisons illustrated in **Tables 1**, **2** with *p*-values of 0.039 and 0.023, respectively.

According to the ACR/EULAR classification criteria for GCA, a score of ≥6 must be obtained for the diagnosis of GCA (5). This can be achieved with or without a positive TA biopsy. The diagnostic clinical score used in our analysis only incorporates the clinical aspects of the ACR/EULAR criteria i.e., the presenting signs and symptoms. The average scores for the biopsy-positive and biopsy-negative groups were 7.5 ± 2.33 and 4.85 ± 2.01 (*p* = 0.003), respectively. This implies that those who were GCA-positive, on average, could have been theoretically diagnosed with GCA based purely on clinical findings as per the above statistically significant difference.

However, keeping in line with the ACR/EULAR considerations, the criteria may only be applied when: a diagnosis of medium or large-vessel vasculitis has been made, when alternate mimickers have been excluded, and finally if the patient’s age was ≥ 50 years at the time of diagnosis as an absolute criterion (5, 18). In our cohort, we have observed an age range of 46 - 93 years, which indicates that the ACR/EULAR considerations for TA biopsy were not strictly followed. When we compared this age range to the corresponding age range in the group of patients with biopsy-confirmed GCA (60 – 81 years), one can conclude that applying the cut-off age of ≥ 50 years for consideration of GCA is reasonable. In addition, the importance of a full clinical exam before any invasive diagnostic measure, i.e., TA biopsy, cannot be understated. Multiple authors have suggested employing clinical algorithms to improve diagnostic protocol (19–21). Some preliminary algorithm models suggest that GCA diagnosis can be achieved through a combination of demographic information, a detailed history, physical exam, and fundoscopic findings (20). We highly recommend developing such an algorithm with stronger consideration for age and clinical manifestations before the performance of a TA biopsy.

Regarding the laboratory investigations, ESR is known to be increased in GCA (15). However, when this parameter was tested in our study, the difference among the analyzed groups (**Tables 1**, **2**) was not statistically significant.

Further efforts to avoid the invasive TA biopsy have been advocated. There is mounting evidence that Doppler ultrasound, as well as magnetic resonance imaging (MRI) or positron emission tomography (PET) scans, should be increasingly utilized in GCA diagnosis (6, 22, 23). Ultrasounds have been shown to have increased sensitivity in detecting GCA in comparison to TA biopsy (6). Thus, the use of ultrasounds may decrease the burden of TA biopsy in various communities, due to an earlier and less invasive diagnosis of GCA. The Rheumatology Spanish Society in their multicenter arteritis study have demonstrated the changes in the diagnostic trends in cases of GCA following the EULAR recommendations (23). Ultrasound use has been increasingly used as a first diagnostic tool in about 53% followed by TA biopsy in 33% then PET scans in 19.6% (23). They also mentioned the impact of the variability in imaging machines and Ultrasonographer’ experience. To our knowledge, none of the patients in this study underwent such imaging, possibly because our Ultrasonographers, who were mainly trained to perform ocular Biomicroscopy, did not have the capacity to perform non-ocular ultrasound studies. Others have advocated the use of MRI as a first imaging tool in their most accurate GCA diagnostic algorithm followed by either US or retinal angiography (14).

Limitations of this study include the small study sample of TA biopsies, where only a small number of patients over several years could have been clinically analyzed retrospectively. This might be related to the relative rarity of GCA in our region of the world, taking into consideration that KKESH is a major referral tertiary eye care center. Despite that, our findings have highlighted important clinical features of GCA that will help guide ophthalmologists to attain better ophthalmic practice when dealing with suspected GCA cases. The sample size for the cases with atherosclerotic changes was even smaller since we only receive the group of these patients who are referred to our eye center or seek ophthalmic consultations because of visual disturbance. Therefore, conducting similar future study in collaboration with a general hospital to recruit higher number of patients in that category and compare their findings to GCA cases would be also beneficial.

## 5 Conclusions

TA biopsy is commonly used for the diagnosis of GCA. However, the incorporation of a unified criteria, targeted history taking, a full head and neck examination, and fundoscopic assessment with certain clinical considerations, can increase the diagnostic accuracy of GCA. It is highly unlikely that biopsies will be rendered completely obsolete but unnecessary biopsies can be avoided by using the ACR/EULAR criteria as a guide. We highly recommend applying the cut-off age of ≥ 50 years as an initial step, followed by the consideration of the following significant clinical features: scalp tenderness, jaw claudication, and ON pallor. Generally, the incorporation of increased clinically focused assessments, imaging techniques, and algorithms, including the clinical scoring that we have extracted from the ACR/EULAR criteria, may decrease the frequency of TA biopsies and prevent unnecessary costs of this procedure. We also advocate performing future prospective multi-center population-based studies on GCA in this part of the world.

## Data availability statement

The raw data supporting the conclusions of this article will be made available by the authors, without undue reservation.

## Ethics statement

The studies involving humans were approved by Human Ethics Committee/Institutional Review Board at King Khaled Eye Specialist Hospital. The studies were conducted in accordance with the local legislation and institutional requirements. The human samples used in this study were acquired from tissue samples that were obtained for diagnostic purpose then included in this retrospective study with expedited approval of the research project. Written informed consent for participation was not required from the participants or the participants’ legal guardians/next of kin in accordance with the national legislation and institutional requirements.

## Author contributions

HA: Conceptualization, Investigation, Methodology, Project administration, Supervision, Writing – original draft, Writing – review & editing. FA: Data curation, Writing – original draft. AM: Investigation, Supervision, Writing – review & editing.
