# Supplementary material for: Giant cell temporal arteritis: a clinicopathological study with emphasis on unnecessary biopsy
Source: Front Ophthalmol (Lausanne). 2023 Dec 6;3:1327420. doi: 10.3389/fopht.2023.1327420 (PMC11182246; doi:10.3389/fopht.2023.1327420)
Supplement: Supplementary file 1 [file Table_1.pdf]

Supplementary Material

1 Supplementary Tables

1.1 Supplementary Table: Summary of ACR/EULAR Classification Criteria for Giant Cell Arteritis (GCA) (5).

Absolute Requirement	*Age ≥ 50 years, at the time of diagnosis*	
Additional Clinical Criteria	Morning stiffness in shoulders and/or neck	+2
	Sudden visual loss	+3
	Jaw or tongue claudication	+2
	New temporal headache	+2
	Scalp tenderness	+2
	Abnormal temporal artery exam*	+2
Laboratory, Imaging, and Biopsy Criteria	Maximum ESR ≥ 50mm/hour or maximum CRP ≥ 10 mg/liter[2]	+3
	Positive temporal artery biopsy or halo sign on temporal artery ultrasound	+5
	Bilateral axillary involvement	+2
	FDG-PET activity throughout aorta	+2
A score of ≥ 6 points is needed for the classification of Giant Cell Arteritis (GCA)		

Supplementary Table 1. Summary of ACR/EULAR Classification Criteria for Giant Cell Arteritis. Consider the following when applying the criteria: 1) Criteria should be applied to classify the patient as having giant cell arteritis when a diagnosis of medium or large-vessel vasculitis has been made, and 2) alternate diagnoses mimicking vasculitis should be excluded. *Includes pulselessness, hard/cord-like texture on palpation, and tenderness
